# Exploring the Association Between Childhood Emotional Maltreatment and Eating Disorder Symptoms During Pregnancy: A Moderated Mediation Model with Prenatal Emotional Distress and Social Support

**DOI:** 10.3390/nu17050902

**Published:** 2025-03-05

**Authors:** Giulia Costanzo, Nadia Barberis, Marco Cannavò, Maria Rita Infurna, Eleonora Bevacqua, Claudia Guarneri, Jada Sottile, Elena Tomba, Giorgio Falgares

**Affiliations:** 1 Department of Psychology, Educational Science, and Human Movement, University of Palermo, 90128 Palermo, Italy; giulia.costanzo01@unipa.it (G.C.); mariarita.infurna@unipa.it (M.R.I.); eleonora.bevacqua@unipa.it (E.B.); claudia.guarneri01@unipa.it (C.G.); jada.sottile@unipa.it (J.S.); 2 Department of Health Sciences, University Magna Graecia of Catanzaro, 88100 Catanzaro, Italy; info@nadiabarberis.it (N.B.); marco.cannavo@studenti.unicz.it (M.C.); 3 Department of Psychology, University of Bologna, 40127 Bologna, Italy; elena.tomba@unibo.it

**Keywords:** childhood emotional maltreatment, eating disorder symptoms, pregnancy, prenatal emotional distress, prenatal social support

## Abstract

Background/Objectives: Pregnancy is a critical period marked by significant transformations that can trigger or exacerbate eating disorder symptoms. Childhood emotional maltreatment, including abuse and neglect, is a known risk factor for disordered eating, yet its specific impact during pregnancy remains unexplored. For this reason, this study aimed to examine the link between childhood emotional maltreatment and eating disorder symptoms in pregnant women, also focusing on the potential mediating and moderating variables involved in this association. Specifically, this study explored the mediating role of prenatal emotional distress, whereas prenatal social support was investigated as a protective factor able to moderate the effects of past trauma on disordered eating during this sensitive period. Methods: Participants were 272 Italian pregnant women (aged 18–48, *M_age_* = 31.21, *SD* = 4.95) who were asked to respond to four self-report instruments: Childhood Trauma Questionnaire—Short Form; Eating Disorder Examination—Questionnaire Short; Perinatal Assessment of Maternal Affectivity; and Maternity Social Support Scale. Results and Conclusions: The results showed that prenatal emotional distress totally mediated the association between childhood emotional maltreatment and eating disorder symptoms in pregnant women (*β* = 0.20; SE = 0.06; 95% CI: 0.08, 0.33; *p* < 0.001). Moreover, moderation analysis showed that prenatal social support only moderated the direct link between childhood emotional maltreatment and disordered eating, so higher levels of childhood emotional maltreatment were predictive of higher levels of eating disorder symptoms only among pregnant women with low levels of prenatal social support (*b* = 0.17; SE = 0.06; *t* = 2.73; 95% CI: 0.05, 0.30; *p* < 0.01). The limitations and clinical implications are discussed.

## 1. Introduction

Eating disorder symptoms refer to a wide range of dysfunctional eating-related behaviors and cognitions, including excessive shape and weight concerns, restricted eating, binge eating, and compensatory behaviors [1]. These disrupted behaviors and cognitions may negatively affect the individual’s physical health, psychosocial functioning, and emotional well-being [2], potentially heightening the risk for a full-blown eating disorder—such as anorexia nervosa, bulimia nervosa, and binge eating disorder [3]. Specifically, according to the Diagnostic and Statistical Manual of Mental Disorders, Fifth Edition (DSM-5) [4], anorexia nervosa is a condition characterized by restricted eating and significant underweight; these symptoms are accompanied by intense fears of gaining weight and altered body image, which strongly affects the individual’s self-evaluation. The influence of body perception on self-evaluation also characterizes bulimia nervosa, which is further described by recurrent episodes of binge eating with feelings of loss of control, typically followed by compensatory behaviors (e.g., vomiting, fasting, use of laxatives, etc.) aimed to prevent weight gain. Finally, binge eating disorder is characterized by recurrent episodes of binge eating occurring in the absence of any compensatory behavior.

It has been suggested that pregnancy is a sensitive period for the onset, reappearance, or worsening of eating disorder symptoms, with prevalence rates comprised between 5.5% and 23.4% for shape and weight concerns, 0.3% and 2.5% for restricted eating, and between 8.4% and 36.1% for binge eating behaviors [1,5]. Specifically, pregnancy poses significant challenges due to physical, psychosocial, and relational changes in a woman’s life. The transition to motherhood requests adjustments to a new role, as well as a redefinition of the relationship with the partner, family, and the whole social environment [6]. Due to these requests, pregnancy may represent a potent stressor that confers vulnerability to emotional suffering and to maladaptive behaviors, such as symptoms of eating disorders, which may be further triggered by difficulties adapting to weight gain and physiological changes in body image [2,5].

Eating disorder symptoms during pregnancy represent an important threat to the health of both the mother and the fetus, as these conditions are associated with poor obstetric outcomes such as an increased risk of miscarriage and preterm delivery, low birth weight, and other postpartum complications [1,7]. Deepening our understanding of the potential risk factors for eating disorder symptoms during pregnancy is, thus, crucial to prevent maternal and fetal morbidity and mortality and to improve the physical and emotional well-being of pregnant women.

### 1.1. Childhood Maltreatment and Eating Disorder Symptoms During Pregnancy

The literature has proposed that a history of traumatic childhood experiences should be conceived as a risk factor for eating disorder symptoms during pregnancy. Specifically, Senior and colleagues [8] found that sexual abuse was a significant predictor of concerns about shape and weight in pregnant women, even after controlling for other risk factors. Moreover, Meltzer-Brody et al. [9] found a high prevalence of sexual and physical abuse experiences among pregnant women with anorexic and bulimic symptoms. However, to the best of our knowledge, no studies have explored the role of emotional forms of maltreatment, such as childhood emotional abuse and childhood emotional neglect, in the onset of eating disorder symptoms during pregnancy.

Childhood emotional abuse refers to a wide range of parental hostile behaviors, comprising verbal aggression, humiliation, and constant criticism toward the child, as well as threats, isolation, and rejection [10]. Childhood emotional neglect, instead, refers to caregivers’ failure to provide emotional support or warmth to the child, along with the inability to respond to their basic psychological needs [11]. Previous studies on the general population have found that childhood emotional abuse and childhood emotional neglect are stronger predictors of eating disorder symptoms as compared to other forms of maltreatment, as suggested by their association with higher scores on different measures of disordered eating (e.g., chronic dieting, overeating, weight and shape concerns, etc.) [12,13,14]. Indeed, emotional abuse and emotional neglect are pervasive forms of maltreatment that stem from a chronically dysfunctional and invalidating family atmosphere, which may impair the child’s ability to tolerate and manage their emotions, above and beyond the effects of other forms of abuse or neglect [15]. This may culminate in symptoms of eating disorders, which may serve as dysfunctional coping strategies to deal with these emotions [14].

Given the unique contribution of emotional abuse and neglect to eating disorder symptoms, addressing this research gap by exploring this association during pregnancy may represent a worthwhile endeavor. At the same time, the mechanisms that could explain this association need further investigation.

### 1.2. Childhood Emotional Maltreatment and Eating Disorder Symptoms During Pregnancy: The Role of Prenatal Emotional Distress

It can be proposed that childhood emotional abuse and neglect predispose to the onset of eating disorder symptoms during pregnancy through their effects on the expectant mother’s emotional well-being.

In line with the attachment theory and the psychodynamic perspective, pregnancy is a period during which women tend to recall memories of their own childhood [16]. When experiences of emotional maltreatment (abuse and neglect) occur in the context of early interactions with caregivers, this reactivation may be emotionally distressing, potentially eliciting emotional suffering in pregnant women [17]. Previous studies have supported this hypothesis, finding that childhood emotional maltreatment was related to conditions of prenatal emotional distress, which embraces a wide range of affective dimensions (e.g., depression, anxiety, irritability/anger, somatization, etc.) [15,18].

In turn, prenatal emotional distress could constitute a risk factor for eating disorder symptoms during pregnancy, as proposed by past research. Specifically, a systematic review by Baskin and Galligan [19] found evidence of a strong relationship between negative affect, particularly symptoms of depression and anxiety, and disordered eating in pregnant women. Disordered eating, indeed, may function as an emotion regulation tool to suppress distressing feelings and momentarily relieve heightened emotional states [5].

However, the mediating role of prenatal emotional distress in the association between childhood emotional maltreatment and eating disorder symptoms during pregnancy has not been explored yet.

### 1.3. Prenatal Social Support as a Protective Factor

It should be noted that not all women with histories of childhood emotional maltreatment develop symptoms of eating disorders later in life. Indeed, some factors may intervene to buffer the detrimental effects of early traumatic experiences on mental health, protecting the individual from the onset of psychopathological conditions.

Particularly during pregnancy, the existence of a nurturing and sensitive interpersonal environment may help an expectant mother deal with pregnancy-activated stressors. Indeed, positive relationships may favor a reframing of negative and distressing beliefs structured during early interactions with caregivers, offering new resources to cope with the challenges of the transition to motherhood [20].

Prenatal social support, conceived as the instrumental and affective involvement of a woman’s interpersonal network (partner, family, and friends) during pregnancy, has been studied as a protective factor for maternal mental well-being, particularly among pregnant women who have experienced childhood maltreatment [21]. Specifically, previous research has found that perceived prenatal support, particularly from a partner, was related to lower negative affect (e.g., depressive symptoms) before childbirth [22,23]. In fact, high levels of social support have the potential to increase perceived coping abilities, lessening the impact of adverse experiences on mental health [24].

To date, there are no studies exploring the buffering effect of prenatal social support in the direct and indirect association between childhood maltreatment and disordered eating. However, it has been observed that pregnant women without eating disorder symptoms reported higher social support from their families as compared to pregnant women with anorexic or bulimic symptoms [25].

Given evidence corroborating the promotive role of social support for pregnant women’s mental health, further exploring its contribution to the interplay between childhood emotional maltreatment, emotional distress, and disordered eating during pregnancy would offer useful information for the prevention of perinatal psychopathological risk and the promotion of the emotional well-being of the expectant mother.

### 1.4. The Present Study

Based on the above considerations, the first aim of the present study was to investigate the association between childhood emotional maltreatment and eating disorder symptoms in a sample of pregnant women, as well as the potential mediating role of prenatal emotional distress in this association. Moreover, the second aim of this study was to test the potential buffering effects of prenatal social support in the direct and indirect association between childhood emotional maltreatment and eating disorder symptoms during pregnancy.

We hypothesized the following:

- Childhood emotional maltreatment would be associated with eating disorder symptoms in pregnancy, and this association would be mediated by prenatal emotional distress (Hypothesis 1);
- Social support would moderate the direct and indirect associations between childhood emotional maltreatment and eating disorder symptoms via prenatal emotional distress (Hypothesis 2).

## 2. Materials and Methods

### 2.1. Participants and Procedure

The Bioethics Committee of the University of Palermo (+/0, ethic approval date: 26 October 2023) approved this study. All the procedures were performed in accordance with the ethical principles for psychological research following the Declaration of Helsinki and its revisions [26] as well as the ethics guidelines of the American Psychological Association [27].

A total of 272 Italian pregnant women (aged 18–48, *M_age_* = 31.21, *SD* = 4.95) participated in the study. The inclusion criteria were (a) being pregnant, (b) being at least 18 years old, (c) being able to understand and speak Italian, and (d) never being diagnosed with an eating disorder. Consequently, the exclusion criteria were (a) not being pregnant, (b) being younger than 18 years old, (c) not being able to understand and speak Italian, and (d) having a current or past diagnosis of eating disorder(s). The participants were recruited from hospitals and birth centers in Southern Italy, from 30 January 2024 to 29 May 2024. All the participants were informed about the research aims, the voluntary nature of participation, and the anonymity of responses. Consent was requested before proceeding with data collection.

Most of the participants were married or cohabitating (87.9%) and primiparous (58.8%). The majority of women (86.4%) reported being in the third trimester of pregnancy. Detailed sociodemographic characteristics and pregnancy-related information are reported in Table 1.

### 2.2. Measures

#### 2.2.1. Childhood Trauma Questionnaire—Short Form

The Italian version of the 28-item Childhood Trauma Questionnaire—Short Form (CTQ-SF) [28,29] was used for the retrospective assessment of childhood experiences of emotional maltreatment. Specifically, this is a self-report questionnaire that evaluates five different types of childhood trauma: emotional abuse, physical abuse, sexual abuse, emotional neglect, and physical neglect. For the purposes of this study, only the “emotional abuse” and the “emotional neglect” subscales were used. To obtain an indicator of emotional maltreatment, the average score of the items comprising these subscales was calculated. All the items were rated on a 5-point Likert scale, ranging from 1 (never true) to 5 (very often true). Higher scores suggest more severe maltreatment experiences.

#### 2.2.2. Perinatal Assessment of Maternal Affectivity

The Perinatal Assessment of Maternal Affectivity (PAMA) [30] is an 11-item self-report instrument used to assess maternal emotional distress during the perinatal period. It comprises 8 items that assess eight different dimensions: anxiety, depression, perceived stress, irritability/anger, relationship problems, psychosomatic reaction, physiological problems (with sleeping or eating), addictions (smoking, taking drugs, and drinking alcohol), and other risky behaviors (such as driving fast after drinking alcohol). The women were asked to rate the severity of each symptom over the last two weeks using a 4-point Likert scale, ranging from 0 (not at all) to 3 (a lot). A total score of maternal emotional distress during the perinatal period was obtained by calculating the average score of these eight dimensions so that higher scores indicate greater perinatal emotional distress. The PAMA also includes 3 additional items related to motherhood; these items were not used to calculate the PAMA total score.

#### 2.2.3. Eating Disorder Examination—Questionnaire Short

The Eating Disorder Examination—Questionnaire Short (EDE-QS) [31] was used to assess the presence of eating disorder symptoms over the last 7 days. This is a 12-item self-report instrument derived from the original 28-item Eating Disorder Examination—Questionnaire [32,33]. Items were rated on a 4-point Likert scale, ranging from 0 to 3. An EDE-QS total score was obtained by calculating the average score of all the items comprising this measure; a higher total score indicates a greater severity of eating disorder symptoms.

#### 2.2.4. Maternity Social Support Scale

The Maternity Social Support Scale (MSSS) [34,35] was used to assess global levels of social support perceived during pregnancy. This self-report questionnaire consists of 6 items that refer to social factors typically associated with perinatal emotional distress (i.e., support from family and friends, help from spouse/partner, conflict with spouse/partner). Items were rated on a 5-point Likert scale, ranging from 1 (never) to 5 (always). Higher scores indicate higher levels of perceived social support.

### 2.3. Data Analysis

Analyses were conducted using IBM SPSS Statistics version 20.0. First, we computed descriptive statistics for the key study variables and reported means (M) and standard deviations (SD). Pearson correlation was used to investigate the possible associations between EDE-QS total score, CTQ emotional maltreatment scores, and the total scores of PAMA and MSSS.

We employed PROCESS macro-Model 4 [36] to test the mediating effect of prenatal emotional distress (PAMA total score) in the relationship between childhood emotional maltreatment experiences (measured via CTQ-SF) and eating disorder symptoms (EDE-QS total score) in pregnancy (Hypothesis 1). Bootstrapping with 5000 resamples was performed to verify the significance of the mediating effect of prenatal emotional distress. If the 95% confidence interval (CI) for the bootstrapped estimate did not include zero, then the indirect effect was considered significant at *p* < 0.05. According to Kenny and Judd [37], indirect effect size estimates around 0.01 are considered small, 0.09 are considered medium, and 0.25 and higher are considered large.

**Figure 1:**
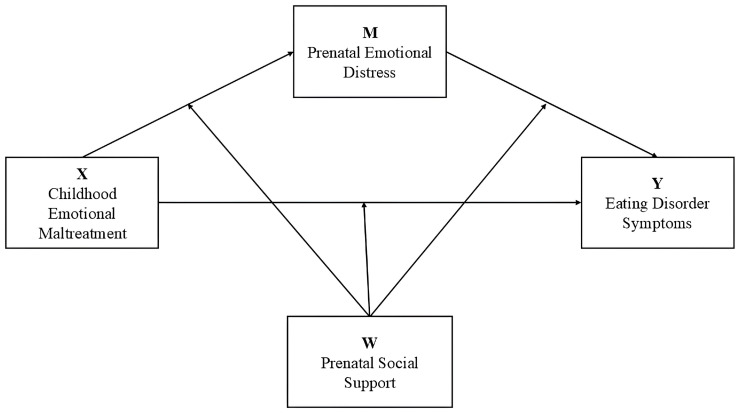
Proposed moderated mediation model.

Finally, through PROCESS macro-Model 59 [36], we run a moderating mediation model to test the moderating role of prenatal social support (MSSS total score; Hypothesis 2) in the indirect and direct effects of childhood emotional maltreatment on eating disorder symptoms in pregnancy (Figure 1). We run bootstrapping with 5000 resamples to test the significance of the hypothesized moderating effects.

## 3. Results

### 3.1. Preliminary Analysis

Means (*M*), standard deviations (*SD*), and correlations among the study variables are presented in Table 2. Eating disorder symptoms were significantly and positively related to childhood emotional maltreatment (*r* = 0.20, *p* < 0.01) and to prenatal emotional distress (*r* = 0.39, *p* < 0.01), whereas significant and negative associations were found with prenatal social support (*r* = −0.14, *p* < 0.05). Prenatal emotional distress was significantly and positively related to childhood emotional maltreatment (*r* = 0.24, *p* < 0.01).

### 3.2. Mediation Analysis

A simple mediation model was tested to explore the relationship between childhood emotional maltreatment (X), prenatal emotional distress (M), and eating disorder symptoms (Y) in pregnancy.

The results revealed a significant effect of childhood emotional maltreatment on prenatal emotional distress (*β* = 0.24; SE = 0.07; 95% CI: 0.15, 0.43; *p* < 0.001), as well as a significant effect of prenatal emotional distress on eating disorder symptoms (*β* = 0.37; SE = 0.05; 95% CI: 0.22, 0.41; *p* < 0.001). The results showed a significant total effect of childhood emotional maltreatment on eating disorder symptoms (*β* = 0.20; SE = 0.06; 95% CI: 0.08, 0.33; *p* < 0.001), as well as a significant indirect effect of childhood emotional maltreatment on eating disorder symptoms through prenatal emotional distress (*β* = 0.09; BootSE = 0.03; 95% CI: 0.04, 0.14). Conversely, the direct effect of childhood emotional maltreatment on eating disorder symptoms in the presence of the mediator was found to be non-significant (*β* = 0.11; SE = 0.06; 95% CI: −0.01, 0.23; *p* = 0.06). This result suggests a total mediation effect of prenatal emotional distress in the relationship between childhood emotional maltreatment and eating disorder symptoms in pregnancy. The model was significant with *R*^2^ = 0.17, *F*(2,269) = 26.84, *p* < 0.001. So, Hypothesis 1 was supported.

### 3.3. Prenatal Social Support as a Moderator

**Figure 2:**
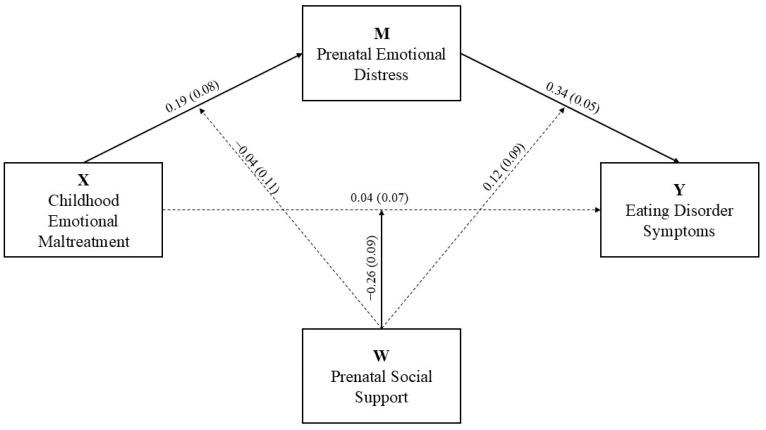
Moderated mediation model with social support proposed as the moderator variable. Note: The values represent the unstandardized coefficients with standard error in parentheses. The solid lines represent the significant paths for *p* < 0.05. The dashed lines represent the non-significant paths.

Figure 2 shows the moderated mediation model with prenatal social support proposed as a moderator variable in the direct and indirect relationship between childhood emotional maltreatment and eating disorder symptoms in pregnancy. The results showed that prenatal social support did not significantly moderate the relationship between childhood emotional maltreatment and prenatal emotional distress and between prenatal emotional distress and eating disorder symptoms.

**Figure 3:**
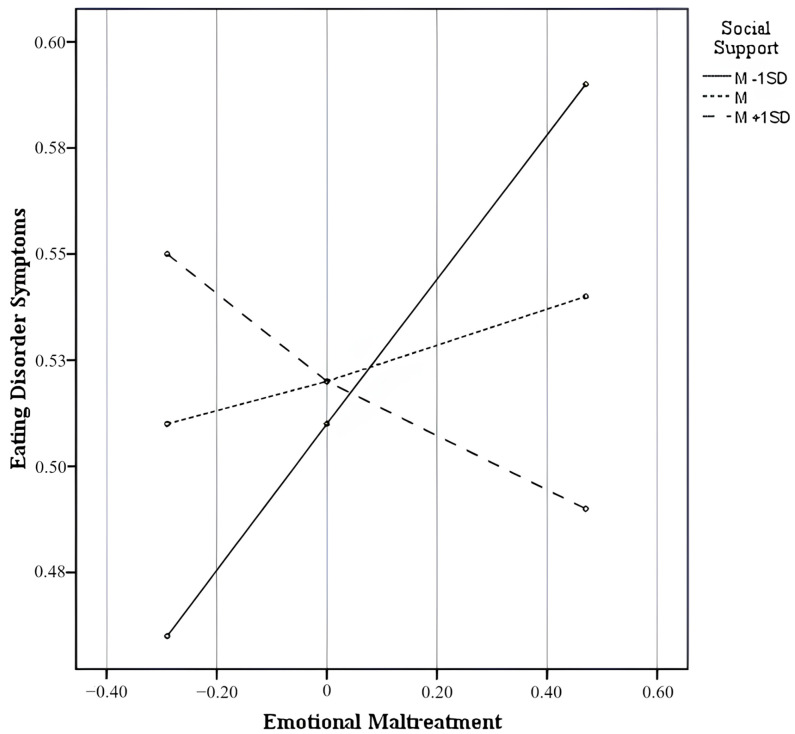
Interaction effects between childhood emotional maltreatment and prenatal social support on eating disorder symptoms.

Only the interaction effect of childhood emotional maltreatment and prenatal social support on eating disorder symptoms was significant, contributing to a significant increase in the explained variance [Δ*R*^2^ = 0.02, *F*(1,266) = 7.87, *p* < 0.01] as suggested by the test of the highest order unconditional interaction. Significant and non-significant interactions are reported in Table 3, along with the conditional direct effects of childhood emotional maltreatment on eating disorder symptoms at different values of the moderator. Specifically, as graphically shown in the slope analysis displayed in Figure 3, the results revealed that higher levels of childhood emotional maltreatment were predictive of higher levels of eating disorder symptoms in pregnancy only among women with low levels of prenatal social support (*b* = 0.17; SE = 0.06; *t* = 2.73; 95% CI: 0.05, 0.30; *p* < 0.01). That is, scarce social support experienced in the prenatal period may exacerbate the detrimental effects of childhood emotional trauma on eating disorder symptoms during pregnancy.

Conversely, the conditional indirect effect of childhood emotional maltreatment on eating disorder symptoms via prenatal emotional distress was not moderated by social support, as confirmed by the bootstrapping results. Specifically, the conditional indirect effect at low, moderate, and high values of social support did not show significant differences, as can be seen in Table 3. Therefore, Hypothesis 2 was only partially supported.

## 4. Discussion

This study aimed to evaluate the relationship between childhood emotional maltreatment and eating disorder symptoms during pregnancy considering the potential mediating role of prenatal emotional distress and the potential moderation effect of prenatal social support in this association.

First, we hypothesized that childhood emotional maltreatment would be associated with eating disorder symptoms during pregnancy and that this association would be mediated by prenatal emotional distress. Our results supported this hypothesis, showing that childhood experiences of emotional maltreatment were related to higher levels of emotional suffering during pregnancy, which in turn were related to higher levels of eating disorder symptoms. These results corroborate the findings from previous studies that showed an association between childhood emotional maltreatment and prenatal emotional distress [15,18] and between prenatal emotional distress and eating disorder symptoms [19].

It should be noted that in this study, emotional distress fully mediated the relationship between childhood adversities and eating disorder symptoms. Although the cross-sectional nature of this study does not allow us to determine causal connections, this result might suggest that early traumatic experiences favor the onset of eating disorder symptoms during pregnancy only through their deleterious effects on the emotional well-being of expectant mothers. This would be in line with psychodynamic theorizations, which propose that the transition to motherhood is a sensitive period during which childhood memories tend to be reactivated and used as references in the creation of a new identity as a mother [20]. When early experiences of emotional maltreatment occurred, this transition could become particularly challenging, as past memories could elicit negative representations of parenthood which can result in increased emotional suffering [17]. Moreover, it is well known that exposure to emotional maltreatment during childhood can lead to impairments in emotion regulation processes [38,39]. Difficulties in identifying, expressing, and managing one’s own emotions could make it more difficult for the expectant woman to deal with trauma-related negative effects, which could, thus, be further exacerbated. This condition of heightened psychological distress, in turn, may favor the use of a wide range of maladaptive coping strategies, including symptoms of eating disorders, which may serve as a tool to regulate or suppress negative emotions and reduce distress [5]. This is in line with previous studies suggesting a relationship between emotion regulation difficulties and eating disorder symptoms [14,40] which could, thus, represent a dysfunctional way to deal with the negative emotional effects exerted by past trauma.

Second, we hypothesized that prenatal social support would moderate the impact of childhood emotional maltreatment on eating disorder symptoms during pregnancy, both directly and indirectly via prenatal emotional distress. The results partially supported our hypothesis, showing that higher levels of childhood emotional maltreatment were directly related to higher levels of eating disorder symptoms in pregnancy only in the presence of low levels of prenatal social support. Conversely, this association was not significant at moderate and high levels of social support. So, it could be suggested that the lack of a supportive interpersonal environment may have the potential to strengthen the deleterious effects of past traumatic experiences on the eating behaviors of the pregnant woman, favoring the onset of eating disorder symptoms.

Some explanations can be proposed. As previously stated, the transition to parenthood poses important challenges, as the woman is requested to adapt to her new role as a mother [6]. As suggested by previous research [21], supportive and caring relationships may represent crucial resources able to foster the woman’s ability to deal with these transformations, which is particularly salient among women with histories of childhood maltreatment. However, when social support from partners, family, and friends is scarce or inadequate, negative representations of self and others formed during early interactions with caregivers can be strengthened [41]; women may experience feelings of loneliness and ineffectiveness in facing maternity-related challenges and tasks, which may increase the risk of maladaptive behaviors, such as eating disorder symptoms, as a way to cope with these overwhelming stressors. Moreover, among several transformations, vulnerable women may encounter more difficulties in adapting to changes in weight and body image, which may represent another trigger for eating disorder symptoms when a supportive and empowering relational environment is not available.

Finally, contrary to our hypothesis, the results showed that prenatal social support did not moderate the association between childhood emotional maltreatment and emotional distress and between emotional distress and eating disorder symptoms. So, it could be proposed that childhood emotional maltreatment exerts its effects on emotional suffering during pregnancy independently from the existence of a supportive network; similarly, the results suggest that support from partners, family, and friends does not protect pregnant women from the onset of eating disorder symptoms as a way to respond to emotional distress. However, other interpersonal factors, such as the quality of communication or the presence of emotional intimacy or involvement with the partner, may play a role in buffering the effects of past trauma on eating disorder symptoms during pregnancy via emotional distress, and thus should be further explored in future research.

### Limitations

Our findings should be considered within the context of some limitations. First, the cross-sectional nature of this research does not allow us to examine causal relationships among the study variables. Future research would benefit from a longitudinal design to determine whether changes in emotional distress and eating disorder symptoms during pregnancy are affected by past experiences of emotional maltreatment, and whether social support from partners, family, and friends buffers this association. Second, we only administered self-report measures, which may be sensitive to social desirability bias, possibly inflating some of the associations among variables. Future research should use a multi-method approach, including qualitative interviews. Third, trying to address the gaps in previous research, the present study only focused on childhood emotional maltreatment, which represents the less explored type of maltreatment in relation to eating disorder symptoms during pregnancy. However, a comparison with childhood physical maltreatment (physical abuse and physical neglect) and childhood sexual abuse would be beneficial and should, thus, be addressed in future research using a larger number of subjects. Fourth, the measure of eating disorder symptoms used in this study, the EDE-QS, was not conceived to specifically evaluate eating disorder symptoms in pregnant women, although its extended version (the EDE-Q) has been widely used in this research field, showing good psychometric properties [42]. Finally, most of the participants in this study were in the third trimester of pregnancy (86.4%), which could bias the results and limit generalizability. Replication of this study with a more heterogeneous sample would foster the generalization of the findings also to women in different stages of pregnancy.

## 5. Conclusions

This study contributes to a better understanding of the relationship between emotional forms of maltreatment and eating disorder symptoms during pregnancy. The results suggest that this association is mediated by prenatal emotional distress, and that it may be reinforced by scarce or inadequate social support from partners, family, and friends.

The findings from this study could have important implications for health care professionals working with pregnant women (gynecologists, nurses, midwives, and psychologists), who should be adequately trained in the recognition of the signs associated with eating disorder symptoms. Indeed, eating disorder symptoms during pregnancy are of clinical concern, as they are associated with a heightened risk of postpartum complications that could represent a threat to the physical and psychological health of both the mother and the baby [1,7]. Comprehensive prenatal screening procedures should incorporate the assessment of potential psychosocial risk factors, such as past traumatic experiences and prenatal signs of psychological suffering, which could predispose the onset of eating disorder symptoms in this sensitive period, in order to identify at-risk women and provide specific and timely interventions aimed to prevent the onset or the exacerbation of these conditions. In particular, evidence from this study suggests that interventions with at-risk women should focus on the revision and resignification of past experiences of emotional maltreatment, with the main goal of favoring the acquisition of a more positive representation of parenthood that could help women dealing with this transition. Moreover, clinical intervention should be directed at strengthening the woman’s ability to manage negative emotions and recur to more adaptive strategies to cope with psychological distress. Finally, professionals should also explore the woman’s perception of support from significant others, as one of the aims of the intervention should be the building of a more positive and caring interpersonal network, which could act as a fundamental resource for the well-being of the expectant mother.

## Figures and Tables

**Table 1:** Sociodemographic and pregnancy-related information.

Variable	n	%
Relationship status		
Married/Cohabitant	239	87.9
Engaged	30	11.0
Divorced	3	1.1
Educational level		
Elementary school diploma	3	1.1
Middle school diploma	53	19.5
High school diploma	89	32.7
Bachelor/Master degree	76	27.9
Post-Lauream degree	51	18.8
Work status		
Housewife	76	27.9
Unemployed	41	15.1
Student	2	0.7
Occasional worker	30	11.0
Employed	123	45.2
Income		
Extremely low	1	0.4
Low	24	8.8
Medium	172	63.2
High	75	27.6
Trimester of pregnancy		
First	5	1.8
Second	32	11.8
Third	235	86.4
Planned pregnancy		
Yes	185	68.0
No	87	32.0
Primiparous		
Yes	160	58.8
No	112	41.2

**Table 2:** Correlation matrix, means, and standard deviations of study variables.

	1	2	3	4
1. Eating Disorder Symptoms	--			
2. Childhood Emotional Maltreatment	0.20 **	--		
3. Prenatal Emotional Distress	0.39 **	0.24 **	--	
4. Prenatal Social Support	−0.14 *	−0.25 **	−0.34 **	--
***M***	0.52	1.29	0.45	4.35
***SD***	0.49	0.47	0.57	0.48

Note: N = 272. * *p* < 0.05; ** *p* < 0.01.

**Table 3:** Moderated mediation analysis with social support as the moderator variable.

*Mediator variable model*				
	Outcome variable: Prenatal Emotional Distress			
	*b* (SE)	*t*	*p*	
Childhood Emotional Maltreatment	0.19 (0.08)	2.39	<0.05	
Prenatal Social Support	−0.35 (0.07)	−4.94	<0.001	
Childhood Emotional Maltreatment x Prenatal Social Support	−0.04 (0.11)	−0.40	NS	
*Dependent variable model*				
	Outcome variable: Eating Disorder Symptoms			
	*b* (SE)	*t*	*p*	
Childhood Emotional Maltreatment	0.04 (0.07)	−0.68	NS	
Prenatal Emotional Distress	0.34 (0.05)	6.34	<0.001	
Prenatal Social Support	0.01 (0.06)	0.21	NS	
Childhood Emotional Maltreatment x Prenatal Social Support	−0.26 (0.09)	−2.81	<0.01	
Prenatal Emotional Distress x Prenatal Social Support	0.12 (0.09)	1.46	NS	
*Conditional direct effect at specific values of the moderator*				
Path	Moderator: Prenatal Social Support	Effect (SE)	LL 95% CI	UL 95% CI
Childhood Emotional Maltratment → Eating Disorder Symptoms	M −1*SD*	0.17 (0.06)	0.05	0.30
	M	0.04 (0.07)	−0.08	0.17
	M +1*SD*	−0.08 (0.09)	−0.27	0.10
*Conditional indirect effect at specific values of the moderator*				
Mediator	Moderator: Prenatal Social Support	Effect (BootSE)	BootLL 95% CI	BootUL 95% CI
Prenatal Emotional Distress	M −1*SD*	0.06 (0.03)	0.02	0.15
	M	0.06 (0.03)	0.02	0.14
	M +1*SD*	0.07 (0.05)	−0.03	0.17
*Pairwise contrasts between conditional indirect effects*				
	Contrast	BootSE	BootLL 95% CI	BootUL 95% CI
Medium vs. Low Prenatal Social Support	0.01	0.02	−0.06	0.04
High vs. Low Prenatal Social Support	0.01	0.05	−0.12	0.08
High vs. Medium Prenatal Social Support	0.00	0.03	−0.07	0.05

## Data Availability

The data presented in this study are available upon reasonable request from the corresponding author.
